# Perceptual consequences of interocular differences in the duration of temporal integration

**DOI:** 10.1167/jov.22.12.12

**Published:** 2022-11-10

**Authors:** Benjamin M. Chin, Johannes Burge

**Affiliations:** 1 Department of Psychology, University of Pennsylvania, Philadelphia, PA, USA; 2 Neuroscience Graduate Group, University of Pennsylvania, Philadelphia, PA, USA; 3 Bioengineering Graduate Group, University of Pennsylvania, Philadelphia, PA, USA

**Keywords:** stereo, temporal integration, motion-in-depth, geometric effect, continuous psychophysics, binding problem

## Abstract

Temporal differences in visual information processing between the eyes can cause dramatic misperceptions of motion and depth. Processing delays between the eyes cause the Pulfrich effect: oscillating targets in the frontal plane are misperceived as moving along near-elliptical motion trajectories in depth (Pulfrich, 1922). Here, we explain a previously reported but poorly understood variant: the anomalous Pulfrich effect. When this variant is perceived, the illusory motion trajectory appears oriented left- or right-side back in depth, rather than aligned with the true direction of motion. Our data indicate that this perceived misalignment is due to interocular differences in neural temporal integration periods, as opposed to interocular differences in delay. For oscillating motion, differences in the duration of temporal integration dampen the effective motion amplitude in one eye relative to the other. In a dynamic analog of the Geometric effect in stereo-surface-orientation perception (Ogle, 1950), the different motion amplitudes cause the perceived misorientation of the motion trajectories. Forced-choice psychophysical experiments, conducted with both different spatial frequencies and different onscreen motion damping in the two eyes show that the perceived misorientation in depth is associated with the eye having greater motion damping. A target-tracking experiment provided more direct evidence that the anomalous Pulfrich effect is caused by interocular differences in temporal integration and delay. These findings highlight the computational hurdles posed to the visual system by temporal differences in sensory processing. Future work will explore how the visual system overcomes these challenges to achieve accurate perception.

## Introduction

Temporal processing changes with the sensory stimuli being processed. Some sensory signals take longer to process than others. Stimulus-based differences in temporal processing delays—relative latencies—have received significant attention in vision science and neuroscience. Luminance signals are processed more quickly than chromatic signals. High luminance signals are processed more quickly than low luminance signals. High contrast signals are processed more quickly than low contrast signals, and low spatial frequencies are processed more quickly than high spatial frequencies. Despite differences in the speed by which these signals are processed, they are integrated by the brain. The computational rules that govern the integration of complementary signals with different temporal dynamics are not yet well understood. Identifying striking perceptual phenomena that result from combining such signals, and developing high-fidelity tools for measuring and characterizing these phenomena, should aid the discovery of computational principles underlying the combination rules.

Binocular integration of information between the eyes is crucial to depth perception. When a scene is viewed binocularly, the images are different in the two eyes because of their different vantage points on the scene. The spatial differences between the images in the two eyes underlie stereopsis, the perception of depth from binocular information. Estimation of these spatial differences can be impacted by differences in temporal processing between the eyes, especially when the images move.

**Figure 1.**
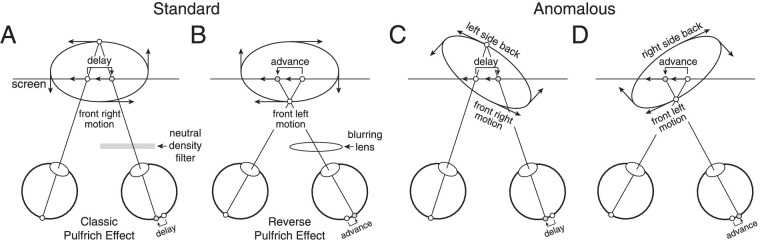
Standard and anomalous versions of the classic and reverse Pulfrich effects. Frontoparallel stimulus motion is misperceived as following near-elliptical trajectories in depth. (**A**) Standard version of the classic Pulfrich effect. A neutral density filter delays the signal in one eye relative to the other by decreasing luminance. (**B**) Standard version of the reverse Pulfrich effect. A blurring lens advances the signal in one eye relative to the other. (**C,** **D**) Anomalous Pulfrich effects. The motion trajectory is perceived as being oriented in depth with respect to the true direction of motion.

Simple processing delays between the eyes cause oscillating targets in the frontal plane to be misperceived as moving along near-elliptical motion trajectories in depth (Figures 1A, B). Such interocular delays cause effective spatial displacements in one eye relative to the other—neural disparities—that result in the illusory motion in depth. This illusion is known as the Pulfrich effect (Pulfrich, 1922). Luminance, contrast, and blur differences between the eyes are all known to cause the effect. Two types of Pulfrich effect have been reported: the classic Pulfrich effect and the reverse Pulfrich effect. In the classic Pulfrich effect, the stimulus in the eye with lower luminance or contrast is processed more slowly (Lit, 1949; Read & Cumming, 2005; Reynaud & Hess, 2017; Wilson & Anstis, 1969; Wolpert, Miall, Cumming, & Boniface, 1993; see Figure 1A). In the reverse Pulfrich effect, the eye with lower image quality (due to blur) is processed more quickly (Burge, Rodriguez-Lopez, & Dorronsoro, 2019; Rodriguez-Lopez, Dorronsoro, & Burge, 2020).

The reverse Pulfrich effect is mediated by blur-induced differences in the spatial frequency content between the stimuli in the two eyes (Burge et al., 2019; Rodriguez-Lopez et al., 2020). Blurring an image low-pass filters the image: high spatial frequencies are selectively removed. Because high spatial frequencies are processed more slowly than low spatial frequencies, the sharp image is processed more slowly than the blurry image. Complementarily, high-pass filtering increases the proportion of high frequencies in the image, and causes the high-pass filtered image to be processed more slowly (Burge et al., 2019). Similarly, if the two eyes are stimulated by moving Gabor stimuli with different carrier frequencies, signals from the eye with higher frequencies are processed more slowly (Min, Reynaud, & Hess, 2020). Thus, simple processing delays (i.e. time shifts in neural responses) nicely account for the standard Pulfrich effect: the perception of illusory 3D motion aligned with the true path of motion.

Anomalous Pulfrich percepts have also been reported (Emerson & Pesta, 1992; Harker & O'neal, 1967; Trincker, 1953; Weale, 1954). In such cases, observers report perceiving near-elliptical motion paths with principal axes that are rotated in depth relative to the true direction of motion (see Figures 1C, D). Simple processing delays cannot explain these percepts. Various explanations have been proposed regarding the cause of anomalous Pulfrich percepts: saccadic suppression, velocity extrapolation, and perceptual distortion of objective visual space (Emerson & Pesta, 1992; Harker & O'neal, 1967; Trincker, 1953; Weale, 1954). But scientific consensus has not coalesced around any of these explanations. This paper clearly explains this previously reported but poorly understood variant of the illusion.

**Figure 2.**
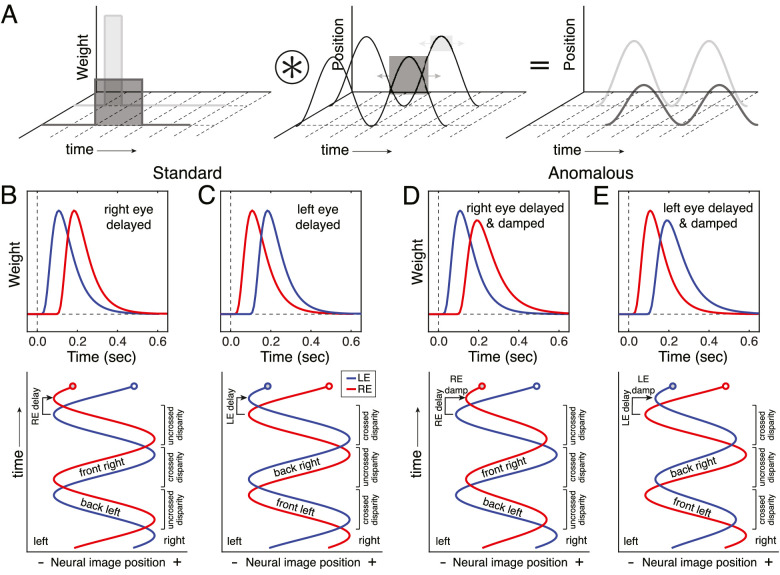
Predicting standard and anomalous Pulfrich percepts. (**A**) The effective position over time (right panel) of a neural image can be modeled as a convolution between an impulse response function (left panel) and the stimulus trajectory (middle panel). Impulse response functions with longer delays and longer temporal integration periods (dark gray) result in longer delays and greater damping of the effective positions over time. Impulse response functions with shorter delays and shorter temporal integration periods (light gray) result in shorter delays and less damping of the effective positions. Shaded squares and arrows in the middle panel indicate the duration of temporal integration and the corresponding range of stimulus positions that are averaged at a particular point in time during the convolution. (**B,** **C**) Temporal impulse response functions (middle row) and effective neural image positions over time (bottom row) for the left eye (blue) and right eye (red) when right-eye processing is delayed relative to left-eye processing, and vice versa. (**D,** **E**) Temporal impulse response functions and left- and right-eye effective neural image positions when right-eye processing is delayed and damped (due to a longer temporal integration period) relative to the left, and vice versa. Standard Pulfrich percepts result from delays only. Anomalous Pulfrich percepts result when the effective motion trajectory in one eye is both delayed and damped relative to the other (see Discussion).

We hypothesize that anomalous Pulfrich percepts—illusory motion trajectories that are rotated in depth with respect to the true direction of motion—are caused by different temporal integration periods; that is, differences in the duration of time over which each eye integrates visual information. To understand this hypothesis, consider the temporal dynamics of sensory processing. The neural response to a sensory stimulus evolves over time. This temporal evolution can be described by an impulse response function. For a moving stimulus, the effective position over time of the neural image in one eye can be modeled as a convolution between the impulse response function and the stimulus trajectory (Figure 2A). The impulse response function can both delay and damp the effective stimulus positions over time relative to the true stimulus positions. The greater the delay of the impulse response function, the greater the delay of the effective neural image positions. The greater the temporal integration period of the impulse response function, the greater the damping of the effective neural image positions. (Note that we use the term “damped” to refer to a fixed reduction in amplitude of a motion signal relative to baseline, rather than progressive attenuation of the amplitude over time.) If the effective trajectory in one eye is delayed relative to that in the other eye (Figures 2B, C), when oscillatory motion is presented stereo-geometry predicts the standard Pulfrich effect (see Figures 1A, B). If the effective trajectory in one eye is both delayed and damped relative to the other eye (Figures 2D, E), stereo-geometry predicts the anomalous Pulfrich effect: illusory motion-in-depth along a trajectory that is misaligned with the true direction of motion (see Figures 1C, D and Discussion).

Informally, we have most often observed anomalous Pulfrich percepts when there is different spatial frequency content in the two eyes. It is well known that different spatial frequencies are processed both with different delays and with temporal integration periods of different durations. Neurons in early visual cortex (V1) and the middle-temporal area (MT) respond to higher spatial frequencies with more delay and longer temporal integration periods, all else equal (Bair & Movshon, 2004; Frazor, Albrecht, Geisler, & Crane, 2004; Vassilev, Mihaylova, & Bonnet, 2002). Psychophysical experiments have shown that the responses of the human visual system as a whole are similarly affected by spatial frequency (Levi, Harwerth, & Manny, 1979).

Here, with a series of three experiments, we test three increasingly detailed aspects of our hypothesis. The first two experiments used a traditional two-alternative forced choice (2AFC) paradigm. Each eye was presented different spatial frequencies and observers reported the perceived orientation of the motion trajectory in depth (left-side back versus right-side back; see Figures 1C, D). Experiment 1 establishes that spatial frequency differences between the eyes can cause the anomalous Pulfrich effect (see Figures 1C, D). Experiment 2 shows that these interocular spatial frequency differences cause differential neural damping of the motion signals in the two eyes (see Figures 2D, E). Experiment 3 gathers evidence, using a recently developed target-tracking paradigm for continuous psychophysics (Bonnen, Burge, Yates, Pillow, & Cormack, 2015), that supports the idea that this differential damping results from different spatial frequencies being processed with different temporal integration periods. We conclude that differences in the duration of temporal integration between the eyes can cause anomalous Pulfrich percepts.

## Results

We conducted three separate experiments to test the hypothesis that mismatched temporal integration periods can cause anomalous Pulfrich percepts, using a within-subject design. The first two experiments used a traditional forced choice paradigm. Observers binocularly viewed an oscillating Gabor stimulus with different carrier spatial frequencies in the two eyes. The task was to indicate the perceived orientation of its motion trajectory in depth. Presenting different images to the two eyes in a task that is supported in part by stereopsis raises concerns about poor binocular fusion. Four of eight screened observers could fuse and perform the forced-choice experiments (see Methods). The third experiment was conducted using continuous target-tracking psychophysics (Bonnen et al., 2015; Bonnen, Huk, & Cormack, 2017; Burge & Cormack, 2020; Knöll, Pillow, & Huk, 2018; Mulligan, Stevenson, & Cormack, 2013). Observers manually tracked a randomly moving Gabor stimulus with a cursor. The results of all experiments support the conclusion that different temporal integration periods can cause differential motion damping in the two eyes, and that this differential damping is the cause of anomalous Pulfrich percepts.

### Experiment 1: Neural damping with mismatched spatial frequencies in the two eyes

Experiment 1 was designed to establish the dependence of the anomalous Pulfrich effect on spatial frequency. On each trial, the observer was dichoptically presented an oscillating Gabor stimulus. The onscreen disparities specified a near-elliptical trajectory in depth that was aligned with the screen.

**Figure 3.**
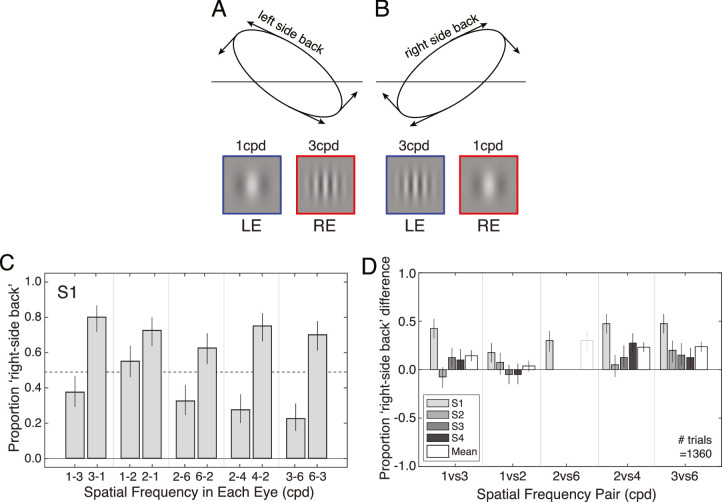
Experiment 1 stimuli, conditions, and results. (**A**) A low frequency Gabor in the left eye and a high frequency Gabor in the right eye predicts that the target will be perceived as moving along a trajectory oriented left-side-back in depth. (**B**) A high frequency Gabor in the left eye and a low frequency Gabor in the right eye predicts that the target will be perceived as moving along a trajectory oriented right-side-back in depth. Although fusion was imperfect, all observers reported percepts of motion in depth. (**C**) Results from one observer. A different spatial frequency was presented to each eye. Each pair of numbers on the x-axis indicates the spatial frequencies presented to the left eye (first number in pair) and right eye (second number in pair). In all cases, right-side-back orientations were reported less often when the low frequency was in the left eye, and more often when the low frequency was in the right eye. (**D**) Results from each individual observer (gray bars) and the mean across observers (white bars; see Methods). The y-axis indicates the difference between the proportion of “right-side-back” responses when the right eye had the lower spatial frequency Gabor versus when the left eye had the lower spatial frequency Gabor (e.g. difference between the 3-1 and 1-3 conditions in **C**). Positive values indicate that right-side-back orientations were reported more often when the lower frequency was in the right eye, and less often when the lower frequency was in the left eye. This pattern held for most conditions and observers. Note that the 2 cpd versus 6 cpd condition is absent from this plot for all but observer S1 (the senior author). In the screening phase (see Methods), no observers other than observer S1 were able to fuse the stimuli in this condition, even when the pattern of onscreen binocular disparities specified a heavily rotated elliptical motion trajectory in depth.

A different carrier spatial frequency was presented to each eye. Under our working hypothesis, the Gabor with the higher spatial frequency should be processed with more delay and (crucially) with a longer temporal integration period than the lower frequency Gabor in the other eye. The longer temporal integration period should cause damping of the effective motion in that eye. The damping, in turn, should cause the illusory orientation of the motion trajectory in depth. We predict that observers will report more left-side-back orientations when the left eye has the lower spatial frequency, and more right-side-back orientations when the right eye has the lower spatial frequency (Figures 3A, B).

Observers were asked to report the apparent orientation of the motion trajectory in depth by indicating with a key press whether the principal axis of the trajectory appeared rotated left-side-back or right-side-back from the plane of the screen. Recall that the onscreen disparities specified that the motion trajectory was aligned with the screen. If spatial frequency differences between the eyes have no effect, the proportion of times observers report left- or right-side-back orientations should be unaffected by which eye is presented the higher spatial frequency. If, on the other hand, the two spatial frequencies are processed with different temporal integration periods, observers should report more right-side-back orientations when the right eye has the lower spatial frequency, and vice versa.

Observers reported more right-side-back orientations of the perceived motion trajectory in depth when the right eye contained the lower frequency and more left-side-back orientations when the left eye contained the lower frequency (Figures 3C, D; also see Supplementary Figure S1). These results implicate interocular differences in spatial frequency as a cause of the anomalous Pulfrich effect.

The null hypothesis is that spatial frequency should have no effect on perceived orientation. That is, the proportion of times observers report right-side-back orientations should not depend on whether the left or right eye was presented the lower spatial frequency. In one observer, the effect appeared in all four conditions. For three observers, the effect was present in three out of the four conditions. We performed binomial tests on the group data to determine whether the null hypothesis could be rejected (see Methods). The tests rejected the null hypothesis at the group level for interocular spatial frequency combinations of 1 cpd versus 3 cpd (*p* < 0.01), 2 cpd versus 4 cpd (*p* < 0.001), and 3 cpd versus 6 cpd (*p* < 0.001), but not 1 cpd versus 2 cpd (*p* = 0.25). At the individual observer level, the proportion of “right-side-back” responses was significantly higher when the right eye was presented the lower spatial frequency for observers S1 (*p* < 0.001) and S3 (*p* = 0.021), but not S2 (*p* = 0.119) and S4 (*p* = 0.063). These results trend in a direction consistent with the experimental hypothesis: that the effective motion signals associated with higher spatial frequencies have amplitudes that are damped due to longer temporal integration periods. But stronger, more direct tests of this hypothesis are possible.

### Experiment 2: Estimating the magnitude of neural damping

**Figure 4.**
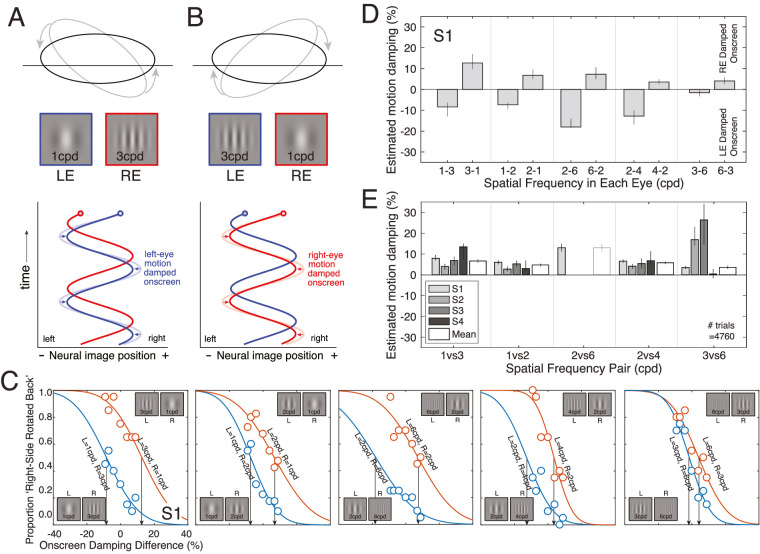
Experiment stimuli, conditions, and results. (**A**) When a higher spatial frequency is presented to the right eye, the effective neural image position trajectory for the right eye (red line) will be damped relative to that of the left eye (light blue line), causing the anomalous Pulfrich effect. However, if the onscreen motion amplitude for the left eye is damped (dark blue line) until the effective neural position trajectories are equal in both eyes, the anomalous Pulfrich effect will be nullified. (**B**) When a higher spatial frequency is presented to the left eye, these patterns are reversed. (**C**) Psychometric functions from the first human observer for five different frequency pairs (i.e. 1 cpd versus 3 cpd, 1 cpd versus 2 cpd, 2 cpd versus 6 cpd, 2 cpd versus 4 cpd, and 3 cpd versus 6 cpd). When the left eye had the lower frequency stimulus, the psychometric functions were shifted to the left (blue points and curves), indicating that the left-eye motion amplitude had to be damped onscreen to null the anomalous component of the Pulfrich effect. When the right eye had the lower frequency stimulus (red points and curves), the psychometric functions were shifted to the right. (**D**) Points of subjective equality (PSEs; arrows in **A**) in each condition for one observer. The PSEs are estimates of the amount of onscreen damping required to null the perceived orientations associated with different spatial frequencies in the two eyes. (**E**) Results from each individual observer (gray bars) and the mean across observers (white bars; see Methods). The average required onscreen motion damping for the lower-frequency stimulus in each spatial frequency pair is shown on the y-axis. Error bars represent bootstrapped ±1 standard errors. Note that the 2 cpd versus 6 cpd conditions are absent for all observers except observer S1. In the screening phase (see Methods), no observers other than observer S1 were able to perform the task in these conditions.

Experiment 2 tested more directly whether presenting a higher spatial frequency to one eye causes the effective motion amplitude in that eye to be reduced (damping; see Figures 2D, E). The logic of the experiment is as follows: if spatial-frequency-induced anomalous Pulfrich percepts are due to neural damping differences, it should be possible to null the anomalous percept by independently manipulating the onscreen motion amplitude in the two eyes (Figures 4A, B). The critical onscreen damping difference should be equal in magnitude but opposite in sign of the neural damping difference induced by the mismatched spatial frequencies in the two eyes.

To measure the onscreen damping difference that nulls the anomalous Pulfrich effect, we directly manipulated onscreen motion amplitude for each eye (Supplementary Experiment 1 confirmed that onscreen differences in motion amplitude change the perceived orientation of the motion trajectory in depth with identical spatial frequencies in the two eyes; Supplementary Figure S2). Observers reported whether the perceived motion trajectory appeared to be oriented left-side-back or right-side-back with respect to the screen.

We collected psychometric data with onscreen damping difference as the independent variable in each condition, and measured the proportion of times that observers reported motion trajectories that were oriented right-side-back with respect to the screen. The data and fitted psychometric functions are shown in Figure 4C (also see Supplementary Figure S3). Consistent with the results of experiment 1, observers reported more “right-side back” responses when the right eye was presented the lower spatial frequency. The effect was highly significant under a binomial test at the group level (*p* < 0.001 for all spatial frequency combinations) as well as at the individual level (*p* < 0.001 for all observers).

The point of subjective equality (PSE) in each condition indicates the onscreen damping that is equal in magnitude and opposite in sign to the corresponding neural damping. When the right eye had the higher spatial frequency stimulus, the left-eye onscreen motion had to be damped to eliminate the anomalous Pulfrich effect, and vice versa (Figure 4D; also see Supplementary Figure S4). This finding held across all tested frequency combinations. The average required onscreen damping for the lower frequency stimulus in each pair of spatial frequencies is shown in Figures 4E*.* These results further support the hypothesis that stimulus-induced differences in temporal integration periods cause neural motion damping that can be neutralized by onscreen motion damping.

### Experiment 3: Continuous target-tracking psychophysics

The results from the first two experiments (i) establish that mismatched spatial frequencies in the two eyes cause anomalous Pulfrich effects, and (ii) show that onscreen damping can eliminate spatial-frequency-induced anomalous Pulfrich effects. Together, these results suggest that different temporal integration periods between the eyes are the root causes of the anomalous Pulfrich effect. The evidence for this conclusion, however, is indirect. To gain more direct evidence that mismatched frequencies induce interocular differences in temporal integration, experiment 3 made use of an entirely different paradigm: continuous target-tracking psychophysics (Bonnen et al., 2015).

**Figure 5.**
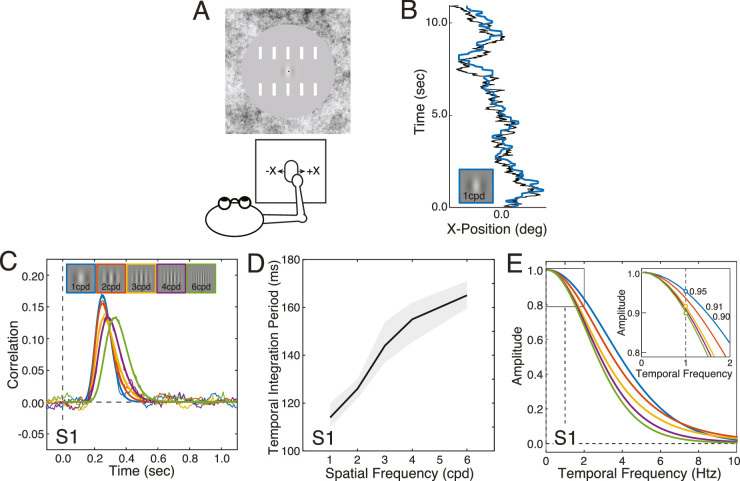
Effects of spatial frequency on target tracking performance for the first human observer. (**A**) On each trial, the observer tracked, with a mouse cursor (black dot at the center of the screen), a Gabor stimulus following a horizontal random walk across the center of the screen. (**B**) Example target-tracking performance on a single trial. The solid black trace indicates the horizontal random walk taken by the stimulus (1 cpd Gabor). The blue trace indicates the position of the observer's cursor. (**C**) Cross-correlograms in the target tracking task derived from target-tracking performance. The cross-correlograms change systematically as a function of spatial frequency (colors). (**D**) The temporal integration period (i.e. full-width at half-height) increases from approximately 115 ms to 165 ms as spatial frequency increases from 1 cpd to 6 cpd. The shaded gray region indicates 68% bootstrapped confidence intervals. (**E**) The amplitude spectra of the cross-correlograms provide an estimate of the amount of effective motion damping for each of many temporal frequencies. The inset shows the estimated amount of visuomotor motion damping for each spatial frequency at 1.0 htz, the temporal frequency of the motion stimulus in the 2AFC experiments.

Using a mouse, observers manually tracked one of five Gabor targets at a time. Each target underwent a horizontal random walk on the screen (Figures 5A, B). The Gabor targets had carrier spatial frequencies of 1 cpd, 2 cpd, 3 cpd, 4 cpd, and 6 cpd. These spatial frequencies were matched to those used in the previous experiments. For example, 1 cpd and 3 cpd spatial frequencies were used in the target-tracking task because conditions in the previous two experiments presented a 1 cpd Gabor to one eye and a 3 cpd Gabor to the other. The tracking task was performed without difficulty. The cross-correlation between the target and response velocities provides information about the temporal processing of the visuomotor system. If the visuomotor system is linear, the cross-correlogram equals an estimate of the temporal impulse response function when the target velocities are white noise, which they are here by design.

The cross-correlograms are broader (and more delayed) in time as spatial frequency increases (Figures 5C, D). These results demonstrate that the visuomotor system processes high spatial frequencies with longer temporal integration periods than low spatial frequencies. The amplitude spectra of the cross-correlograms can be used to determine the proportion by which the motion amplitude associated with each spatial frequency is damped as a function of temporal frequency (Figure 5E, see Methods). The inset in Figure 5E shows that at 1.0 htz—the temporal frequency at which targets oscillated in the 2AFC forced-choice experiments (i.e. experiments 1 and 2)—the motion amplitude of the visuomotor response in the tracking task decreases as spatial frequency increases. (The same is true for other temporal frequencies.) In other words, the amplitude of the visuomotor response is increasingly damped as target spatial frequency increases.

If the motor component of the visuomotor response does not change with spatial properties of the stimulus (e.g. spatial frequency), then changes in the visuomotor response should reflect only changes in visual processing. In this case, changes in visual processing should be faithfully preserved in the movement of the hand. Evidence for the preservation of visual processing changes have been reported for both the movement of the hand (Burge & Cormack, 2020), and for smooth pursuit eye movements (Lee, Joshua, Medina, & Lisberger, 2016). If (i) damping of the visuomotor response reflects damping of the visual response, and (ii) damping of the visual response is implicated in the anomalous Pulfrich effect, then the visuomotor tracking responses (experiment 3; see Figure 5E) should predict the magnitude of the anomalous Pulfrich effect (experiment 2; see Figure 4), and vice versa. We plotted estimates of damping derived from the two experiments against each other.

**Figure 6.**
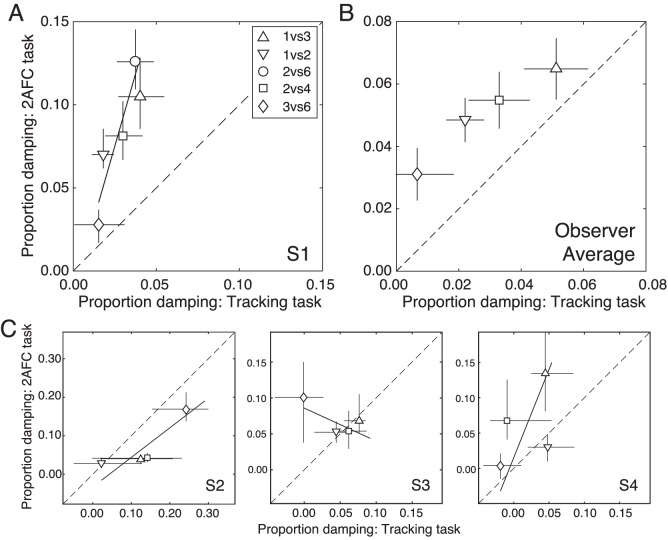
Comparison of 2AFC-based versus target-tracking-based estimates of motion damping. (**A**) Results for the first observer. The best-fit line via weighted linear regression (solid), and the unity line (dashed) are also shown. Error bars on data points indicate 68% bootstrapped confidence intervals. (**B**) Average results across all observers (see Methods). (**C**) Results for each of the other three observers.

For the first human observer, the damping estimates are strongly correlated (*r* = 0.90, *p* < 0.04; Figure 6A). The group average shows a similar trend (*r* = 0.98, *p* = 0.02; Figure 6B). For all observers but one, the same qualitative pattern exists: sensory-perceptual- and tracking-based estimates of motion damping increase together. However, the slopes of the best-fitting lines vary substantially across observers (Figure 6C). On an observer-by-observer basis, it will therefore be difficult to predict estimates of visual motion damping in the forced-choice task from the magnitude of the visuomotor motion damping in the tracking task, or vice versa (see Discussion). Nevertheless, the results are largely consistent—at the group level and at the individual observer level—with the hypothesis that effective motion damping underlies anomalous Pulfrich effects.

## Methods

### Participants

Four human observers participated in the experiment. Three observers were male and one observer was female. One was an author and the rest were naïve to the purposes of the experiment. All had normal or corrected to normal visual acuity (20/20), and normal stereoacuity as determined by the Titmus Stereo Test. The observers were aged 23, 26, 27, and 42 years old at the time of the measurements. All observers provided informed consent in accordance with the Declaration of Helsinki using a protocol approved by the Institutional Review Board at the University of Pennsylvania.

### Apparatus

Stimuli were presented on a custom four-mirror stereoscope. Left- and right-eye images were presented on two identical Vpixx VIEWPixx LED monitors. The monitors were 52.2 × 29.1 cm, with a spatial resolution of 1920 × 1080 pixels, a refresh rate of 120 Hz, and a maximum luminance of 105.9 cd/m^2^. After light loss due to mirror reflections, the maximum luminance was 93.9 cd/m^2^. The gamma function of each monitor was linearized using custom software routines. A single AMD FirePro D500 graphics card with 3GB GDDR5 VRAM controlled both monitors to ensure that the left and right eye images were presented simultaneously. To overcome bandwidth limitations of the monitor cables, custom firmware was written so that a single color channel drove each monitor; the red channel drove the left monitor and the green channel drove the right monitor. The single-channel drive to each monitor was then split to all three channels for gray scale presentation.

Observers viewed the monitors through a pair of mirror cubes positioned one inter-ocular distance apart. The mirror cubes had 2.5 cm openings. Given the eye positions relative to the openings, the field of view through the mirror cubes was approximately 15 × 15 degrees. The outer mirrors were adjusted such that the vergence distance matched the 100 cm distance of the monitors. This distance was confirmed both by a laser ruler measurement and by a visual comparison with a real target at 100 cm. At this distance, each pixel subtended 1.09 arcmin. Stimulus presentation was controlled via the Psychophysics Toolbox-3 (Brainard, 1997). Anti-aliasing enabled sub-pixel resolution permitting accurate presentations of disparities as small as 15 to 20 arcsec. Heads were stabilized with a chin and forehead rest.

### Forced-choice psychophysics: Temporal stimulus properties

For the forced-choice psychophysics experiments, the stimulus oscillated left and right on the display screen. For each trial, the left- and right-eye onscreen Gabor positions in degrees of visual angle were given by

(1a) $$x_{L}\left(t\right)=E_{L}\cos\left(2\pi \omega \cdot \left(t+\Delta t\right)+\phi_{0}\right)$$

(1b) $$x_{R}\left(t\right)=E_{R}\cos\left(2\pi \omega \cdot \left(t\right)+\phi_{0}\right)$$

where *E_L_* and *E_R_* are the left- and right-eye motion amplitudes in degrees of visual angle, Δ*t* is the onscreen delay between the left- and right-eye target images, ω is the temporal frequency of the target movement, ϕ_0_ is the starting phase, and *t* is time in seconds.

The undamped motion amplitude was 1.5 degrees of visual angle (3.0 degrees total change in visual angle in each direction). The maximum onscreen motion damping in one eye (20%) corresponded to 80% (1.2 degrees of visual angle) of the undamped amplitude in the other. The range of particular damping values was adjusted to the sensitivity of each observer. The onscreen interocular delays were set at ±25 ms. The temporal frequency was 1 cycle per second, and the presentation duration was 1 second. The starting phase ϕ_0_ was randomly chosen on each trial to equal either 0 or π, which forced the stimuli to start either to the left or to the right of the center.

When the onscreen interocular difference in motion amplitude equals zero and the onscreen interocular delay is zero, the target moves in the frontoparallel plane at the distance of the screen; the onscreen disparities are zero throughout the trial. If the interocular difference in motion amplitude is non-zero and/or if the interocular delay is non-zero, spatial binocular disparities result and the disparity-specified target follows a motion-in-depth trajectory outside the plane of the monitor. Differences in motion amplitude cause a disparity-specified misalignment in depth of the motion trajectory. Non-zero delays cause a disparity-specified elliptical trajectory of motion in depth. Negative delay values indicate the left-eye onscreen image is delayed relative to the right; positive delay values indicate the left eye onscreen image is advanced relative to the right.

The onscreen binocular disparity for a given interocular delay and damping as a function of time is given by

(2) $$\begin{array}{ccc} \;\Delta x\left(t\right)\; & = & x_{R}\left(t\right)-x_{L}\left(t\right)\\ & = & \sqrt{E_{L}^{2}+E_{R}^{2}-2E_{L}E_{R}\cos\left(2\pi \omega \cdot \Delta t\right)}\cos\left(\phi_{0}\right)\\ & & \times \;\;\cos\left[2\pi \omega t-\mathrm{ta}n^{-1}\left[\frac{E_{L}\sin\left(2\pi \omega \cdot \Delta t\right)}{E_{R}-E_{L}\sin\left(2\pi \omega \cdot \Delta t\right)}\right]\right]\; \end{array}$$

where negative disparities are crossed (i.e. nearer than the screen) and positive disparities are uncrossed (i.e. farther than the screen). The disparity takes on its maximum magnitude when the perceived stimulus is directly in front of the observer and the lateral movement is at its maximum speed. The maximum disparity in visual angle occurs when t= ta n-1[EL sin (2πω·Δt)ER-EL sin (2πω·Δt)]/2πω and is given by Δxmax=EL2+ER2-2ELER cos (2πω·Δt). Note that to induce a desired onscreen delay, we did not actually temporally manipulate when left- and right-eye images were presented onscreen. Both eyes’ images were presented simultaneously on each monitor refresh. Rather, from the desired onscreen delay and the stimulus velocity on each time step, we calculated the equivalent onscreen disparity $\Delta x=\dot{x}\Delta t$ and appropriately shifted the spatial positions of the left- and right-eye images.

Two sets of five vertically oriented picket-fence bars (0.25 × 1.00 degrees) flanked the region of the screen traversed by the target stimulus. The picket fences were specified by disparity to be at the screen distance. A 1/f noise texture, also defined by disparity to be at the screen distance, covered the periphery of the display. Both the picket fences and the 1/f noise texture served as stereoscopic references to the screen distance and helped to anchor vergence.

Before the target appeared on each trial, a small dot appeared either 1.5 degrees to the left of center or 1.5 degrees right of center, where the target was about to appear. Observers were instructed to fixate on the dot and then, after the target appeared, fixate and follow the target throughout the trial. In pilot experiments, we found that if observers did not follow the target with their eyes, the highest spatial frequency (i.e. 6 cpd) Gabor occasionally appeared to vanish during the trial at and near its maximum velocity. At the end of a trial, the stimulus disappeared, and observers reported whether the perceived motion trajectory was oriented left-side-back or right-side-back from frontoparallel. Observers could take as long as they wished to respond. All experiments used a one-interval, 2AFC procedure.

### Forced-choice psychophysics: Spatial stimulus properties

Vertically oriented Gabor stimuli were presented in the forced-choice psychophysical experiments. A vertically oriented Gabor is given by the product of a sinewave carrier and a Gaussian envelope:

(3) $$G\left(x,y\right)=gauss\left(x,y;\sigma_{x},\sigma_{y}\right)\cos\left(2\pi fx+\phi \right)$$

where σ_*x*_ and σ_*y*_ are the standard deviation in X and Y of the Gaussian envelope, *f* is the frequency of the carrier, and ϕ is the phase. Five Gabor targets with different carrier frequencies were used: 1 cpd, 2 cpd, 3 cpd, 4 cpd, and 6 cpd. All had the same spatial size because all had the same Gaussian envelope (σ_*x*_ = 0.39 and σ_*y*_ = 0.32). The octave bandwidths thus equaled 1.5, 0.7, 0.46, 0.35, and 0.23 and the orientation bandwidths equaled 60 degrees, 32 degrees, 22 degrees, 16 degrees, and 11 degrees, respectively. The phase of the carrier frequency was equal to 0.0 for all Gabor stimuli (i.e. all Gabors were in cosine phase).

Experiments 1 and 2 both presented Gabors with different spatial frequencies in the two eyes. Data were collected in blocks with an intermixed design. Conditions in which the left eye was stimulated with the lower spatial frequency (e.g. 1 cpd to the left eye, and 3 cpd to the right eye; see Figure 3A) were interleaved with conditions in which the right eye was stimulated with the lower spatial frequency (e.g. 3 cpd to the left eye, and 1 cpd to the right eye; see Figure 3B). There are two benefits to this design. First, idiosyncratic block-specific response biases that may be present on a given block should be equally distributed amongst both conditions and have little effect on the final results. Second, because humans are poor at utrocular discrimination, it is difficult to determine which of the two eyes are being presented a given stimulus (Blake & Cormack, 1979; Schwarzkopf, Schindler, & Rees, 2010). Intermixing conditions ensures that, on any given trial, observers were unclear about which eye was being presented which stimulus. Hence, it would be quite difficult for observers to deliberately respond in a manner consistent with the experimental hypothesis.

Experiment 2 was designed to measure full psychometric functions in each condition using the method of constant stimuli. Data were collected at seven levels of onscreen damping in each condition. The psychometric functions were fit with a cumulative Gaussian via maximum likelihood methods. The 50% point on the psychometric function—the PSE—indicates the onscreen motion damping needed to null the relative motion damping due to spatial frequency differences. Observers ran 140 trials per condition (i.e. 140 trials per psychometric function) in counter-balanced blocks of 70 trials each.

Experiment S1 presented Gabors with the same spatial frequency in the two eyes. Two onscreen interocular delays (±25 ms) and two onscreen interocular damping levels (% ±5) were presented. We chose these values so that the orientation of the near-elliptical 3D motion trajectories (left-side-back versus right-side-back) were easy for the observers to identify.

### Observer screening

In the screening phase, observers were shown mismatched spatial frequencies in the two eyes (1 cpd versus 3 cpd) with a large amount of onscreen damping (20%). This amount of damping far exceeded the relative neural damping caused by the frequency differences, thus ensuring that the onscreen damping should determine the perceived orientation of the trajectory in depth. If an observer did not correctly report the direction of the stereo-specified misalignment at least 80% of the time, no further data were collected from that observer. Four out of eight screened observers were excluded from the study on this basis. The excluded observers all reported difficulty fusing and difficulty seeing any stereo-specified depth at all. The pilot data are consistent with these reports.

### Binomial test for significance

Under our working hypothesis, “right-side back” responses should be reported more often when the effective motion-amplitude in the left eye is smaller than that in the right eye. Similarly, when the effective motion-amplitude in the right eye is smaller than that in the left eye, “right-side back” responses should be reported less often. The null hypothesis predicts that there will be no difference in the proportion of “right-side back” responses across two matched conditions (e.g. 1 cpd versus 3 cpd and 3 cpd versus 1 cpd). To determine whether the proportions of “right-side back” responses differed significantly from those predicted by the null hypothesis, we used a binominal test. Under the null hypothesis, the probability, *p*, of the observed response proportions is given by

(4) $$\;p=\sum_{i=0}^{n-k}\left[\left(\begin{array}{c} n\\ i \end{array}\right)\pi_{0}^{i}{\left(1-\pi_{0}^{i}\right)}^{n-i}\sum_{j=i+k}^{n}\left(\begin{array}{c} n\\ j \end{array}\right)\pi_{0}^{j}{\left(1-\pi_{0}^{j}\right)}^{n-j}\right]$$

where *n* is the number of trials in a given condition, π_*o*_ is the probability of the observer responding “right-side back” in each of the two matched conditions under the null hypothesis (i.e. the observed mean of the two matched conditions), and *k* is the difference in the number of “right-side back” responses between the two matched conditions. We computed this probability (Equation 4) both at the group level (with trials combined across observers) and at the individual observer level (with trials combined across spatial frequency conditions, i.e. combining all conditions in which the left eye was presented the lower spatial frequency, and combining all conditions in which the right eye was presented the lower spatial frequency).

### Reliability-weighted averaging of estimated motion damping

PSEs estimates in experiment 2 were averaged across observers using reliability-weighted averaging:

(5a,b) $$\begin{array}{c} \hat{D}=\sum_{i=1}^{N}r_{i}{\hat{D}}_{i}/\sum_{i=1}^{N}r_{i},\;r=1/s^{2},\; \end{array}$$

where $\hat{D}$ is the estimate of motion damping for a given condition, averaged across all observers. *N* is the number of observers and *s* is the standard error of motion damping estimates (as determined by 68% bootstrapped confidence intervals). Reliability-weighted averaging takes into consideration differences in the reliability of damping estimates across observers. These differences in reliability arise because some observers are more sensitive to onscreen motion damping than others. It is well-known from signal detection theory that greater sensitivity in a task is associated with more reliable estimates of the point of subjective equality (here, estimates of motion damping).

### Estimated relationship between forced-choice- and target-tracking-based motion damping estimates

The relationship between 2AFC-based and target-tracking-based estimates of motion damping was fit with a line via weighted linear regression. Because estimates of motion damping from both tasks have associated uncertainty, simple linear regression is not appropriate. This is because simple linear regression assumes that one of the variables is independent, and thus has no associated uncertainty. We fit the parameters of the best-fit line with maximum likelihood methods using numerical optimization. The cost function was

(6) $$\begin{array}{ccc} \;c & = & \sum_{i=1}^{N}\left[{\left({\hat{D}}_{tracking,i}-{\bar{D}}_{tracking,i}\right)}^{2}/\sigma_{tracking,i}^{2}\right]\\ \; & & +\sum_{i=1}^{N}\left[{\left({\hat{D}}_{2AFC,i}-a-b{\bar{D}}_{tracking,i}\right)}^{2}/\sigma_{2AFC,i}^{2}\right]\; \end{array}$$

where *N* is the total number of conditions for an observer, $\hat{D}$ is the experiment-derived estimate of motion damping for a given condition, $\bar{D}$ is a free parameter indicating the expected amount of motion damping for a given condition, σ is the standard error of the motion damping estimate for a given condition (as determined by 68% bootstrapped confidence intervals), *a* is the y-intercept of the best fit line, and *b* is the slope of the best fit line.

### Target-tracking procedure

Tracking data were collected from each observer in blocks of individual runs. Each run was initiated with a mouse click, which caused the target and a small dark mouse cursor to appear in the center of the screen. After a stationary period of 500 ms, the target began a one-dimensional horizontal random walk (i.e. Brownian motion) for 11 seconds. The task was to track the target as accurately as possible with a small dark mouse cursor. Blocks contained intermixed runs from each of the four conditions.

### Target-tracking psychophysics: Spatial stimulus properties

Data were collected in five conditions, each of which was distinguished by a different target Gabor stimulus. Each Gabor target had one of five different carrier frequencies: 1 cpd, 2 cpd, 3 cpd, 4 cpd, and 6 cpd. All Gabor targets shared the same Gaussian envelope (σ_*x*_ = 0.39 degrees and σ_*y*_ = 0.32 degrees), and subtended approximately 2.0 degrees × 2.0 degrees of visual angle (i.e. five sigma). Hence, in the five conditions, the octave bandwidths equaled 1.5, 0.7, 0.46, 0.35, and 0.23 and the orientation bandwidths equaled 60 degrees, 32 degrees, 22 degrees, 16 degrees, and 11 degrees, respectively. Data were collected in five intermixed blocks of 20 runs each for a total 20 runs per condition.

### Target-tracking psychophysics: Temporal stimulus properties

For the tracking experiments, the target stimulus performed a random walk on a gray background subtending 10.0 × 7.5 degrees of visual angle, and was surrounded by a static field of 1/f noise. The region of the screen traversed by the target was flanked by two horizontal sets of 13 vertically oriented picket fence bars (see Figure 5A).

The x-positions of the target on each time step *t* + 1 were generated as follows

(7) $$x\left(t+1\right)=x\left(t\right)+{ɛ}_{x}\;;\;{ɛ}_{x}∼N\left(0,Q\right)$$

where ε_*x*_ is a random sample of Gaussian noise and *Q* is the drift variance. The random sample determines the change in target position between the current and the next time step. The drift variance determines the expected magnitude of the position change on each time step, and hence the overall variance of the random walk. The variance of the walk positions across multiple walks σ^2^(*t*) = *Qt* is equal to the product of the drift variance and the number of elapsed time steps. The value of the drift variance in our task (0.8mm per time step) was chosen to be as large as possible such that each walk would traverse as much ground as possible while maintaining the expectation that less than one walk out of 500 (i.e. less than one per human observer throughout the experiment) would escape the horizontal extent of the gray background area (176 × 131 mm) before the 11 second trial completed.

The effective onscreen positions of the images are obtained by convolving the onscreen target image positions with the temporal impulse response function

(8) $$\tilde{x}\left(t\right)=x\left(t\right)*h\left(t\right)$$

where *h*(*t*) is a temporal impulse response function corresponding to a specific frequency. Convolving the target velocities with the impulse response function gives the velocities of the effective target images. Integrating these velocities across time gives the effective target positions.

To estimate the impulse response function relating the target and response, we computed on each trial the zero-mean normalized cross-correlation between the target and response velocities

(9) $$\begin{array}{ccc} \;\rho \left(\tau ;\dot{x},\dot{\tilde{x}}\right) & = & \frac{1}{∥\dot{x}\left(t\right)∥∥\dot{\tilde{x}}\left(t\right)∥}\\ \; & & \times \;\left[\sum_{t=1}^{N}\left(\dot{x}\left(t\right)-\dot{\mu }\right)\left(\dot{\tilde{x}}\left(t+\tau \right)-\dot{\tilde{\mu }}\right)\right]\; \end{array}$$

where τ is the lag, $\dot{x}$ and $\dot{\tilde{x}}$ are the target and response velocities, and $\dot{\mu }$ and $\dot{\tilde{\mu }}$ are the mean target and mean response velocities on the trial. To compute the normalized cross-correlations, we did not include the first second of each 11-second tracking run so that observers had reached steady state tracking performance (although leaving the first second in does not materially change the results). The mean cross-correlation functions shown in the figures were obtained by first computing the normalized cross-correlation in each run (Equation 9), and then averaging these cross-correlograms across runs in each condition.

Assuming a linear system, when the input time series (i.e. the target velocities) is white, as it is here by design, the cross-correlation with the response gives the impulse response function of the system. Another implication of a linear system is that if the impulse response kernel has a sum of coefficients equal to 1.0, then observer responses must have a gain equal to 1.0. The fact that observer responses in our data have gains very close to 1.0 (see Supplementary Figure S6) justifies the normalization of the cross-correlations in Equation 9.

### Gamma distribution fits to mean cross-correlograms

To summarize the mean cross-correlograms, we fit a Gamma function using maximum likelihood methods. The form of the fitted function was given by

(10) $$\begin{array}{ccc} \rho \left(\tau \right) & = & A\left[1/\left(\Gamma \left(s\right)m^{s}\right)\right]{\left(\tau -d\right)}^{s-1}\\ & & \times \;\exp\left[-\left(\tau -d\right)/m\right] \end{array}$$

where *A* is the amplitude, and *m*, *s*, and *d* are the parameters determining the shape and scale of the fit. The mode (i.e. peak) of the function is given by *ms*. The full-width at half-height is used as a measure of the temporal integration period, and is computed via numeric methods. The damping associated with a given fitted function is given by the value of the normalized amplitude spectrum at the temporal frequency of the stimulus, which in the current experiments is one cycle per second.

## Discussion

In this paper, we presented evidence that anomalous Pulfrich percepts—illusory motion trajectories in depth misaligned with the true direction of motion—are caused by interocular differences in temporal integration periods in the two eyes. This specific perceptual effect, and the reasons it occurs, have more general implications.

The integration of multiple complementary streams of incoming information with different temporal dynamics is fundamental to the performance of biological systems. In most cases, sensory-perceptual systems successfully solve this temporal binding problem, and compute accurate estimates of environmental properties. In some cases, the visual system fails to compensate for temporally mismatched signals, and inaccurate estimates result. Such cases are instructive. They can help reveal fundamental properties about the temporal nature of sensory signals, and make plain the striking perceptual consequences of insufficient compensatory mechanisms.

In this Discussion section, we contextualize the anomalous Pulfrich effect with reference to other areas of vision research, consider how visual and visuomotor measures of performance are related, and discuss potential future directions.

### Analogy to the geometric effect in surface orientation perception

**Figure 7.**
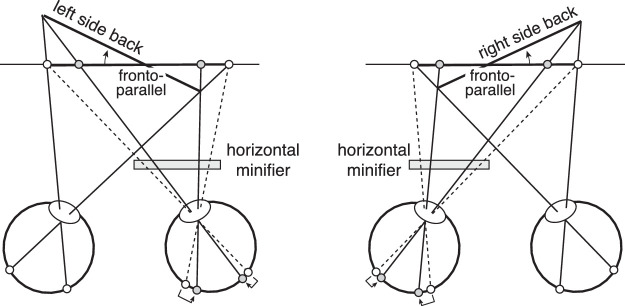
The Geometric effect in stereo-slant perception. Horizontal minification (or magnification) distorts the pattern of binocular disparities such that the disparity-specified orientation of the surface appears rotated in depth. If the horizontal minifier is in front of the right eye, a frontal surface straight-ahead is perceived left-side-back. If the horizontal minifier is in front of the left eye, a frontal surface straight-ahead is perceived right-side-back. The same principles account for both the Geometric effect and the anomalous Pulfrich effect.

Horizontal minification (or magnification) of the image in one eye causes the misperception of surface orientation. This phenomenon is known as the Geometric effect (Banks & Backus, 1998; Ogle, 1950). The Geometric effect occurs because the horizontal minification in one eye distorts the patterns of binocular disparity such that they specify a surface slant that is different from the actual surface slant. For example, when a frontoparallel surface is viewed with a horizontal minifier in front of the right eye, the surface is perceived to be slanted left-side-back. If the left-eye image is minified, the same surface is perceived to be slanted right-side-back (Figures 7).

The principles behind the Geometric effect mirror the principles behind the anomalous Pulfrich effect. An obvious analogy can be drawn between right- or left-eye motion damping and right- or left-eye horizontal minification. Anomalous Pulfrich percepts are caused by motion that is differentially damped between the two eyes. Indeed, if the effective image motion is damped but not delayed in one eye relative to the other, the disparity-specified motion trajectory lies in the plane of the slanted surface specified by disparities caused by the Geometric effect.

### Preservation of sensory processing dynamics in motor movements

The current paper reports a series of results that strongly suggest that different spatial frequencies are processed with different temporal integration periods (and temporal delays), and that these differences underlie the anomalous Pulfrich effect. However, it is important to keep in mind that making inferences about sensory-perceptual processing from the target-tracking results requires an assumption. The assumption is that changes in the ability of an observer to track a target across different target stimuli reflect changes in the sensory-perceptual processing of the stimuli as opposed to changes in the motor response. Multiple studies have shown that this assumption holds in various situations. Motor variation in smooth-pursuit eye movements is due overwhelmingly to sensory errors (Osborne, Lisberger, & Bialek, 2005). Changes in the width of the cross-correlogram associating target and hand movements during target-tracking are linked to the sensitivity of visual target location discrimination (Bonnen et al., 2015). Delays in visual processing match delays in the motor response of both the eye (Lee et al., 2016), and the hand (Burge & Cormack, 2020; Lee et al., 2016). However, it appears from the present experiments that differences in the visual temporal integration period are not always faithfully preserved in the motor response of the hand.

Experiments 1 and 2 used traditional forced-choice psychophysical techniques to establish the anomalous Pulfrich phenomenon and quantify the effective motion damping caused by different spatial frequencies. Experiment 3 used continuous target-tracking psychophysics to collect more direct evidence that different spatial frequencies are indeed associated with different temporal integration periods. The average estimates of motion damping across human observers from the target-tracking task very nearly matched those from the forced-choice task (see Figure 6B). But there was significant interobserver variability regarding how the two sets of estimates were related (see Figures 6A, C). In two of four observers, the forced-choice-based estimates were systematically larger than the tracking-based estimates. In one observer, the reverse was true. And in the last observer, the estimates were nearly matched, except for an apparent outlier.

The finding that forced-choice- and target-tracking-based estimates of damping are correlated but do not exactly agree for individual observers warrants further study. There are several avenues one might consider pursuing. Our analysis assumes that the motor component of the visuomotor response can be accurately modeled with convolution, a linear open-loop computation. It is likely that there are benefits to modeling visuomotor performance in the target-tracking task as a closed-loop system, given that visual feedback is integral to good performance in many visuomotor tasks. It is also possible that convolution does not accurately capture how the motor system translates visual input into a motor response. Control-theoretic frameworks that incorporate feedback and/or movement costs, or other (possibly nonlinear) operations might be required to accurately model the motor contribution to performance. Additionally, the forced-choice task required that the left- and right-eye images be binocularly combined, whereas the tracking task did not. This discrepancy may contribute to the variability in the data. These, and related, issues are under active investigation.

### Computational challenges of mismatched temporal processing

The visual system must constantly deal with the problem of staggered information arrival. We have focused on the perceptual consequences of temporal processing differences associated with mismatched spatial frequency content in the two eyes. Interocular differences in spatial frequency content commonly occur in natural viewing. During binocular viewing of surfaces that are slanted about a vertical axis, for example, the spatial frequencies tend to be higher in one eye than the other. These differences, although extremely common, tend to be relatively small. For a surface at a distance of 30 cm and a slant of 72 degrees, the corresponding frequencies in the two eyes will differ by approximately a factor of two (i.e. horizontal size ratios of 0.5 or 2.0, depending on whether the surface is slanted left- or right-side back). For more distant and less slanted surfaces, which are more common in natural viewing (Adams, Elder, Graf, Leyland, Lugtigheid, & Muryy, 2016; Backus, Banks, van Ee, & Crowell, 1999; Burge, McCann, & Geisler, 2016; Kim & Burge, 2018; Kim & Burge, 2020; Yang & Purves, 2003), the ratio tends to be substantially smaller. However, typical natural images have broadband 1/f spectra, and frequencies above the contrast detection threshold typically vary by a factor of 10 or more. Thus, the temporal binding problem may be a more acute computational challenge within each eye's image than between the images in the two eyes. In spite of this challenge, the visual system usually generates (largely) accurate estimates of environmental properties.

Measuring the temporal processing constraints of the nervous system, and developing normative theory for how different streams of information should be integrated for accurate perception, will help advance our understanding of how the spatial-frequency binding problem is resolved by biological systems (Burge et al., 2019). Incorporating these solutions into image-computable ideal observers for sensory-perceptual tasks with natural stimuli is a potentially fruitful future direction for neuroscience and vision research (Burge, 2020; Burge & Geisler, 2011; Burge & Geisler, 2012; Burge & Geisler, 2014; Burge & Geisler, 2015; Chin & Burge, 2020).

## Conclusion

The problem of binding temporally damped and temporally staggered information is not a niche problem. It is not specific to the combination of information from different spatial frequency channels, as we have focused on in this paper. The visual system must resolve temporal differences between luminance and various chromatic signals, high and low luminance signals, and high and low contrast signals. More generally, the different senses—visual, auditory, vestibular, proprioceptive, and tactile—transmit signals possessing substantially different temporal properties. These signals must be combined to form accurate, temporally coherent percepts. Future work will investigate how sensory-perceptual systems solve the temporal binding problem within and across senses.

## Supplementary Material

Supplement 1

## References

[bib1] Adams, W. J., Elder, J. H., Graf, E. W., Leyland, J., Lugtigheid, A. J., & Muryy, A. (2016). The Southampton-York Natural Scenes (SYNS) dataset: Statistics of surface attitude. *Scientific Reports,* 6, 35805. 27782103 10.1038/srep35805 PMC5080654

[bib2] Backus, B. T., Banks, M. S., van Ee, R., & Crowell, J. A. (1999). Horizontal and vertical disparity, eye position, and stereoscopic slant perception. *Vision Research,* 39(6), 1143–1170. 10343832 10.1016/s0042-6989(98)00139-4

[bib3] Bair, W., & Movshon, J. A. (2004). Adaptive temporal integration of motion in direction-selective neurons in macaque visual cortex. *Journal of Neuroscience,* 24(33), 7305–7323. 15317857 10.1523/JNEUROSCI.0554-04.2004 PMC6729763

[bib4] Banks, M. S., & Backus, B. T. (1998). Extra-retinal and perspective cues cause the small range of the induced effect. *Vision Research,* 38(2), 187–194. 9536348 10.1016/s0042-6989(97)00179-x

[bib5] Blake, R., & Cormack, R. H. (1979). On utrocular discrimination. *Perception & Psychophysics,* 26, 53–68. 10.3758/bf03202005 7335447

[bib6] Bonnen, K., Burge, J., Yates, J., Pillow, J., & Cormack, L. K. (2015). Continuous psychophysics: Target-tracking to measure visual sensitivity. *Journal of Vision,* 15(3), 1–16. 10.1167/15.3.14 PMC4371613 25795437

[bib7] Bonnen, K., Huk, A. C., & Cormack, L. K. (2017). Dynamic mechanisms of visually guided 3D motion tracking. *Journal of Neurophysiology,* 118(3), 1515–1531. 28637820 10.1152/jn.00831.2016 PMC5596126

[bib8] Brainard, D. H. (1997). The psychophysics toolbox. *Spatial Vision,* 10(4), 433–436. 9176952

[bib9] Burge, J. (2020). Image-computable ideal observers for tasks with natural stimuli. *Annual Review of Vision Science,* 6, 491–517. 10.1146/annurev-vision-030320-041134 32580664

[bib10] Burge, J., & Cormack, L. K. (2020). Target tracking reveals the time course of visual processing with millisecond-scale precision. *bioRxiv* *Preprint*. 10.1101/2020.08.05.238642.

[bib11] Burge, J., & Geisler, W. S. (2011). Optimal defocus estimation in individual natural images. *Proceedings of the National Academy of Sciences,* 108(40), 16849–16854. 10.1073/pnas.1108491108 PMC3189032 21930897

[bib12] Burge, J., & Geisler, W. S. (2012). Optimal defocus estimates from individual images for autofocusing a digital camera (Vol. 8299, p. 82990E). Presented at the Proceedings of the IS&T/SPIE 47th Annual Meeting, Burlingame, CA: Proceedings of SPIE.

[bib13] Burge, J., & Geisler, W. S. (2014). Optimal disparity estimation in natural stereo images. *Journal of Vision,* 14(2), 1–18. 10.1167/14.2.1 PMC3912897 24492596

[bib14] Burge, J., & Geisler, W. S. (2015). Optimal speed estimation in natural image movies predicts human performance. *Nature Communications,* 6, 7900. 10.1038/ncomms8900 PMC4532855 26238697

[bib15] Burge, J., McCann, B. C., & Geisler, W. S. (2016). Estimating 3D tilt from local image cues in natural scenes. *Journal of Vision,* 16(13), 2. 10.1167/16.13.2 PMC5066913 27738702

[bib16] Burge, J., Rodriguez-Lopez, V., & Dorronsoro, C. (2019). Monovision and the Misperception of Motion. *Current Biology,* 29(15), 2586–2592.e4. 31353183 10.1016/j.cub.2019.06.070 PMC6730667

[bib17] Chin, B. M., & Burge, J. (2020). Predicting the partition of behavioral variability in speed perception with naturalistic stimuli. *Journal of Neuroscience,* 40(4), 864–879. 31772139 10.1523/JNEUROSCI.1904-19.2019 PMC6975300

[bib18] Emerson, P. L., & Pesta, B. J. (1992). A generalized visual latency explanation of the Pulfrich phenomenon. *Perception & Psychophysics,* 51(4), 319–327. 1603645 10.3758/bf03211625

[bib19] Frazor, R. A., Albrecht, D. G., Geisler, W. S., & Crane, A. M. (2004). Visual cortex neurons of monkeys and cats: temporal dynamics of the spatial frequency response function. *Journal of Neurophysiology,* 91(6), 2607–2627. 14960559 10.1152/jn.00858.2003

[bib20] Harker, G. S., & O'neal, O. L. (1967). Some observations and measurements of the Pulfrich phenomenon. *Perception & Psychophysics,* 2, 438–440.

[bib21] Kim, S., & Burge, J. (2018). The lawful imprecision of human surface tilt estimation in natural scenes. *eLife,* 7, e31448. 29384477 10.7554/eLife.31448 PMC5844693

[bib22] Kim, S., & Burge, J. (2020). Natural scene statistics predict how humans pool information across space in surface tilt estimation. *PLoS Computational Biology,* 16(6), e1007947–e1008026. 32579559 10.1371/journal.pcbi.1007947 PMC7340327

[bib23] Knöll, J., Pillow, J. W., & Huk, A. C. (2018). Lawful tracking of visual motion in humans, macaques, and marmosets in a naturalistic, continuous, and untrained behavioral context. *Proceedings of the National Academy of Sciences,* 115, E10486–E10494. 10.1073/pnas.1807192115 PMC6217422 30322919

[bib24] Lee, J., Joshua, M., Medina, J. F., & Lisberger, S. G. (2016). Signal, Noise, and Variation in Neural and Sensory- Motor Latency. *Neuron,* 90(1), 165–176. 26971946 10.1016/j.neuron.2016.02.012 PMC4824642

[bib25] Levi, D. M., Harwerth, R. S., & Manny, R. E. (1979). Suprathreshold spatial frequency detection and binocular interaction in strabismic and anisometropic amblyopia. *Investigative Ophthalmology & Visual Science,* 18(7), 714–725. 447470

[bib26] Lit, A. (1949). The magnitude of the Pulfrich stereophenomenon as a function of binocular differences of intensity at various levels of illumination. *The American Journal of Psychology,* 62(2), 159–181. 18131264

[bib27] Min, S. H., Reynaud, A., & Hess, R. F. (2020). Interocular Differences in Spatial Frequency Influence the Pulfrich Effect. *Vision,* 4(20), 1–13. 10.3390/vision4010020 PMC7157571 32244910

[bib28] Mulligan, J. B., Stevenson, S. B., & Cormack, L. K. (2013). Reflexive and voluntary control of smooth eye movements. In B. E. Rogowitz, T. N. Pappas, & H. de Ridder (Eds.), (Vol. 8651, pp. 86510Z1–22). Presented at the IS&T/SPIE Electronic Imaging XVIII, SPIE.

[bib29] Ogle, K. N. (1950). *Researches in binocular vision*. Philadelphia, London: W B Saunders.

[bib30] Osborne, L. C., Lisberger, S. G., & Bialek, W. (2005). A sensory source for motor variation. *Nature,* 437(7057), 412–416. 16163357 10.1038/nature03961 PMC2551316

[bib31] Pulfrich, C. (1922). Die Stereoskopie im Dienste der isochromen und heterochromen Photometrie. *Die Naturwissenschaften,* 10(35), 553–564.

[bib32] Read, J. C. A., & Cumming, B. G. (2005). Effect of interocular delay on disparity-selective v1 neurons: relationship to stereoacuity and the Pulfrich effect. *Journal of Neurophysiology,* 94(2), 1541–1553. 15788521 10.1152/jn.01177.2004 PMC1414116

[bib33] Reynaud, A., & Hess, R. F. (2017). Interocular contrast difference drives illusory 3D percept. *Scientific Reports,* 7(1), 5587. 28717190 10.1038/s41598-017-06151-w PMC5514099

[bib34] Rodriguez-Lopez, V., Dorronsoro, C., & Burge, J. (2020). Contact lenses, the reverse Pulfrich effect, and anti-Pulfrich monovision corrections. *Scientific Reports,* 10, 1–16. 32999323 10.1038/s41598-020-71395-y PMC7527565

[bib35] Schwarzkopf, D. S., Schindler, A., & Rees, G. (2010). Knowing with which eye we see: utrocular discrimination and eye-specific signals in human visual cortex. *PLoS One,* 5(10), e13775. 21048942 10.1371/journal.pone.0013775 PMC2966441

[bib36] Trincker, D. (1953). Brightness-darkness adaptation and special vision. I. Phenomenology of the Pulfrich's effect with reference to asymmetry-phenomenon. *Pflugers Archiv Fur Die Gesamte Physiologie Des Menschen Und Der Tiere,* 257(1), 48–69. 10.1007/BF00363411 13099963

[bib37] Vassilev, A., Mihaylova, M., & Bonnet, C. (2002). On the delay in processing high spatial frequency visual information: reaction time and VEP latency study of the effect of local intensity of stimulation. *Vision Research,* 42(7), 851–864. 11927350 10.1016/s0042-6989(01)00300-5

[bib38] Weale, R. A. (1954). Theory of the Pulfrich effect. *Ophthalmologica. Journal International D'ophtalmologie. International Journal of Ophthalmology. Zeitschrift Fur Augenheilkunde,* 128(6), 380–388. 10.1159/000302399 14356707

[bib39] Wilson, J. A., & Anstis, S. M. (1969). Visual delay as a function of luminance. *The American Journal of Psychology,* 82(3), 350–358. 5350004

[bib40] Wolpert, D. M., Miall, R. C., Cumming, B., & Boniface, S. J. (1993). Retinal adaptation of visual processing time delays. *Vision Research,* 33(10), 1421–1430. 8333163 10.1016/0042-6989(93)90048-2

[bib41] Yang, Z., & Purves, D. (2003). Image/source statistics of surfaces in natural scenes. *Network (Bristol, England),* 14(3), 371–390. 12938763

