# Supplementary material for: Perceptual consequences of interocular differences in the duration of temporal integration
Source: J Vis. 2022 Nov 10;22(12):12. doi: 10.1167/jov.22.12.12 (PMC9652723; doi:10.1167/jov.22.12.12)
Supplement: Supplement 1 [file jovi-22-12-12_s001.pdf]

Supplement:
Perceptual consequences of interocular differences in the duration of temporal integration

Benjamin M. Chin[1.] & Johannes Burge[1.,2.,3.]

[1.] Department of Psychology, University of Pennsylvania
[2.] Neuroscience Graduate Group, University of Pennsylvania
[3.] Bioengineering Graduate Group, University of Pennsylvania

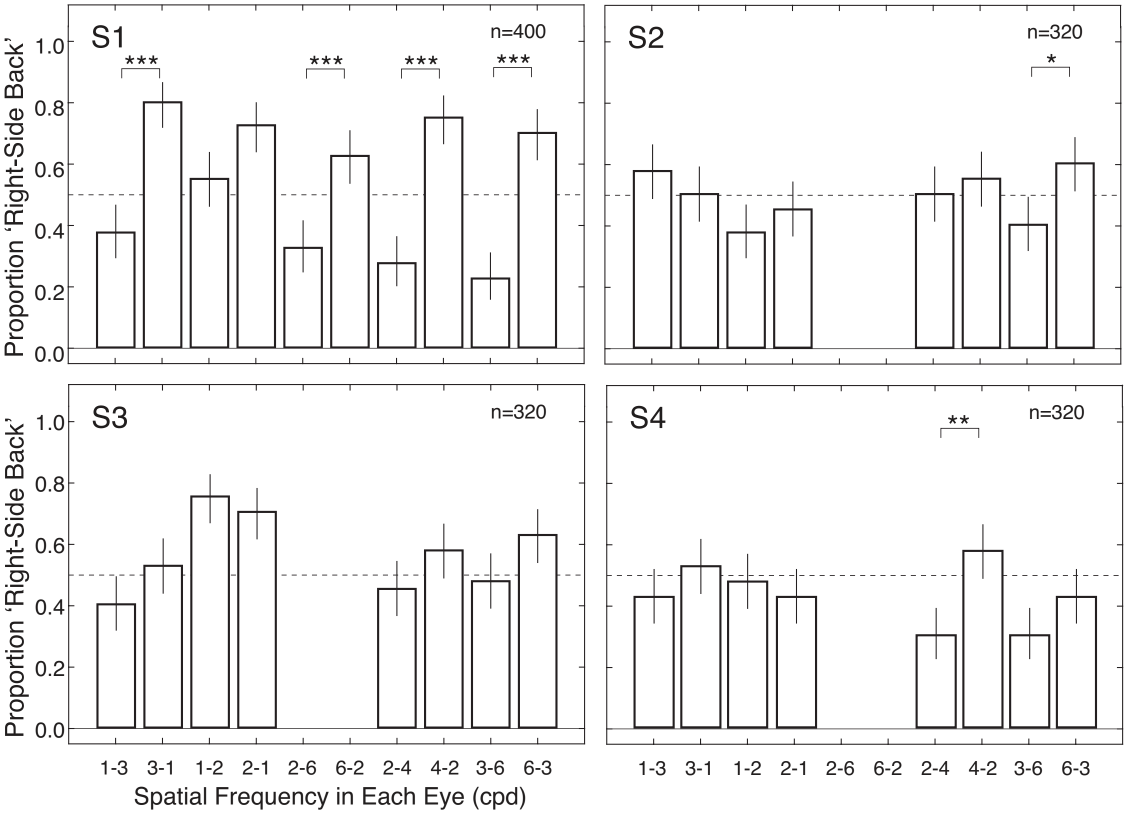

Figure S1. Experiment 1 results for all observers and conditions. All plots have the same format as Figure 3C. Asterisks with brackets indicate statistical significance for pairs of conditions from each observer based on a binomial test (1 asterisk indicates $p<0.05$; 2 asterisks indicate $p<0.01$; 3 asterisks indicate $p<0.001$).

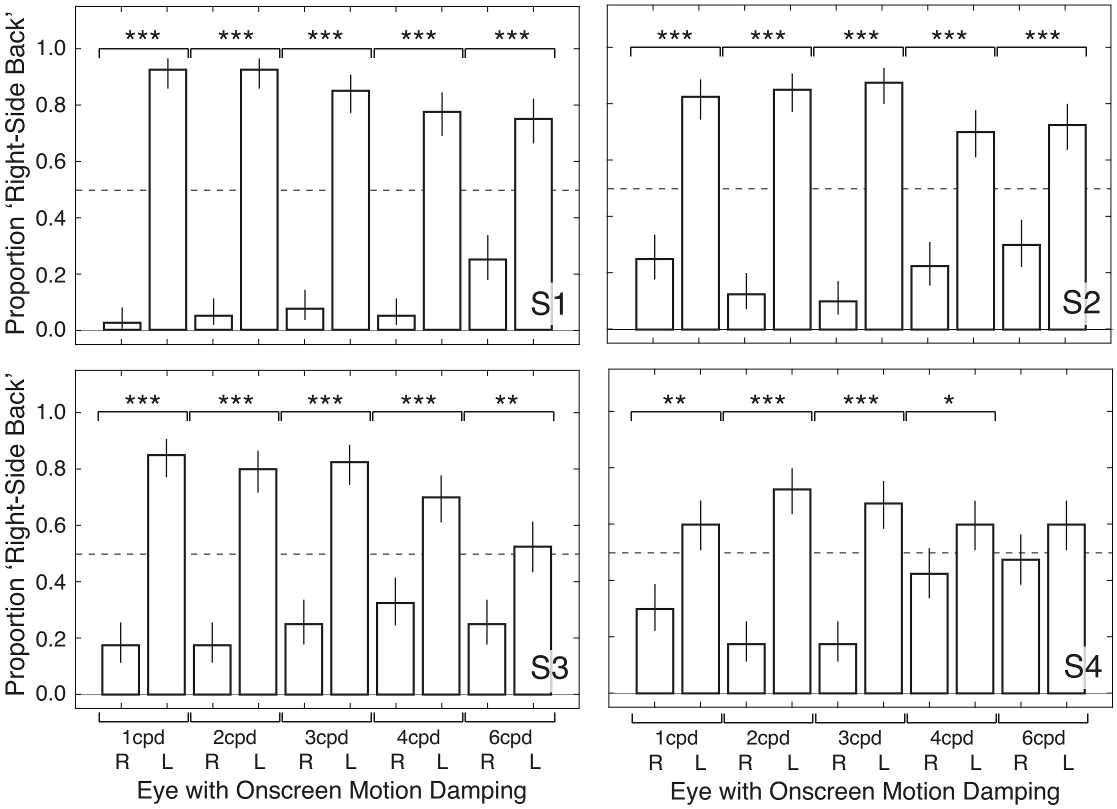

Figure S2. Supplementary Experiment 1 stimuli, conditions, and results. When the eyes are presented Gabors with the same spatial frequency, but the right-eye motion amplitude is damped (and delayed) onscreen, stereo-geometry specifies a near-elliptical motion trajectory that is oriented left-side back with respect to the screen. When the eyes are presented Gabors with the same spatial frequency, but the left-eye motion amplitude is damped (and delayed) onscreen, stereo-geometry specifies a near-elliptical motion trajectory that is oriented right-side back with respect to the screen. Results from all observers are shown. For all frequencies, responses are consistent with stereo-geometry-based predictions. Error bars indicate ±1 standard error. Asterisks with brackets indicate statistical significance for pairs of conditions from each observer based on a binomial test (1 asterisk indicates $p<0.05$; 2 asterisks indicate $p<0.01$; 3 asterisks indicate $p<0.001$). Results from Experiment S2 are stronger and more consistent than those in Experiment 1. This is not surprising, since the stimuli in Experiment S2 are have identical spatial patterns between the eyes and are thus easier to fuse.

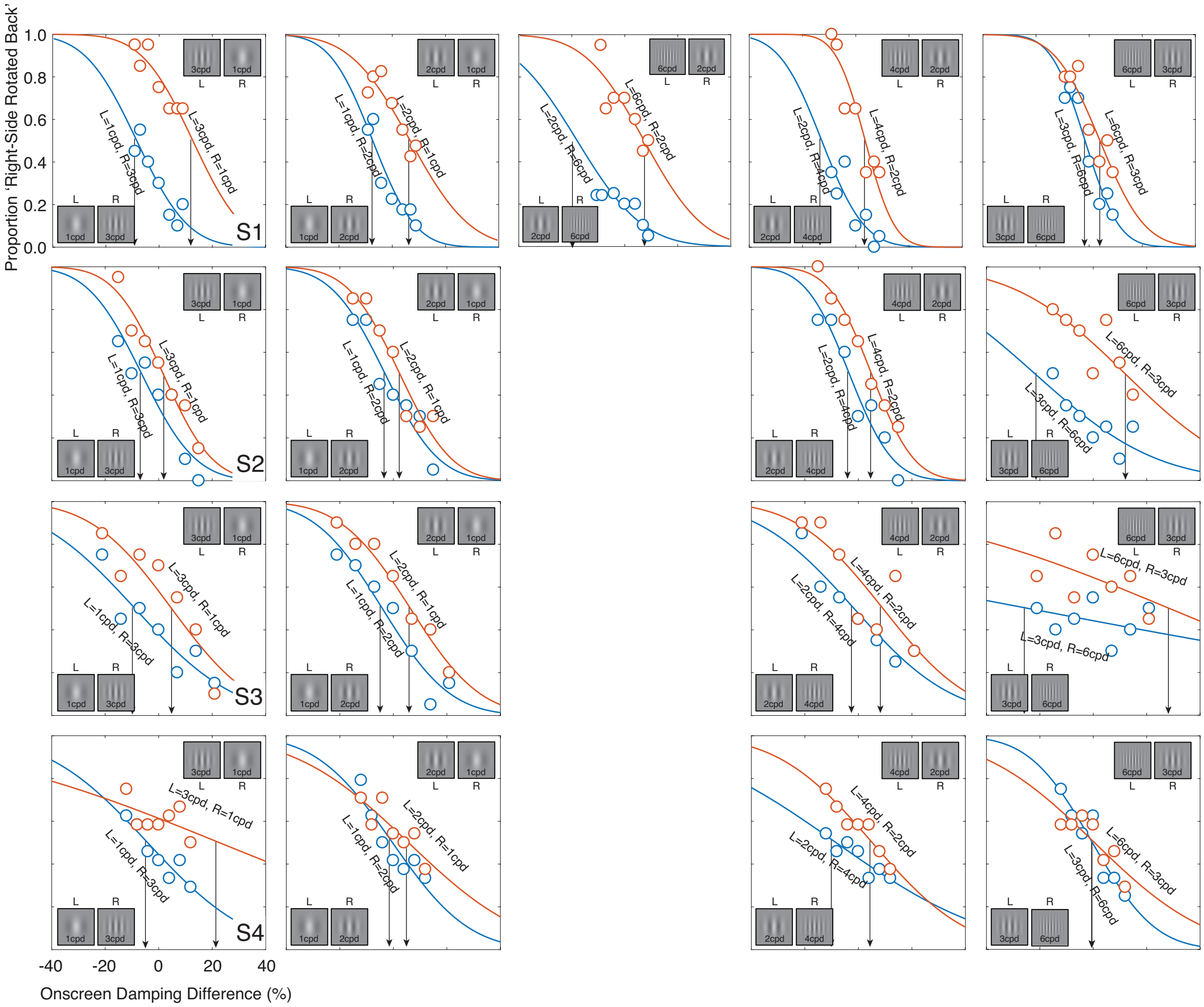

Figure S3. Experiment 3 stimuli, conditions, and psychometric functions for all observers and conditions. The data from all four observers follow the same qualitative pattern.

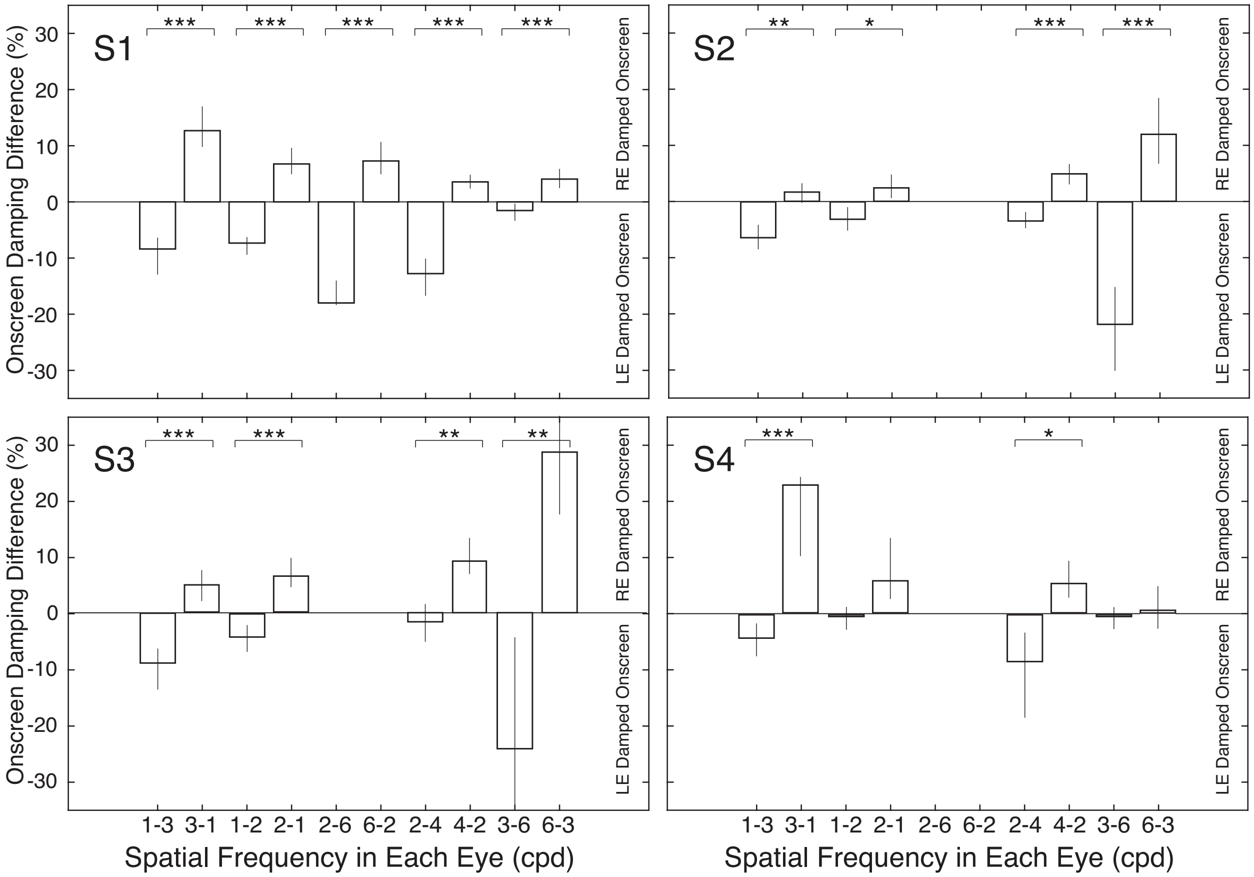

Figure S4. Points of subjective equality (PSEs; arrows in Fig. S3) for all observers and conditions. The PSEs are estimates of the amount of onscreen damping required to null the perceived orientations associated with different spatial frequencies in the two eyes. The data from all four observers follow the same qualitative pattern. Asterisks with brackets indicate statistical significance for individual pairs of conditions from each observer based on bootstrapping of the PSE values (1 asterisk indicates $p<0.05$; 2 asterisks indicate $p<0.01$; 3 asterisks indicate $p<0.001$).

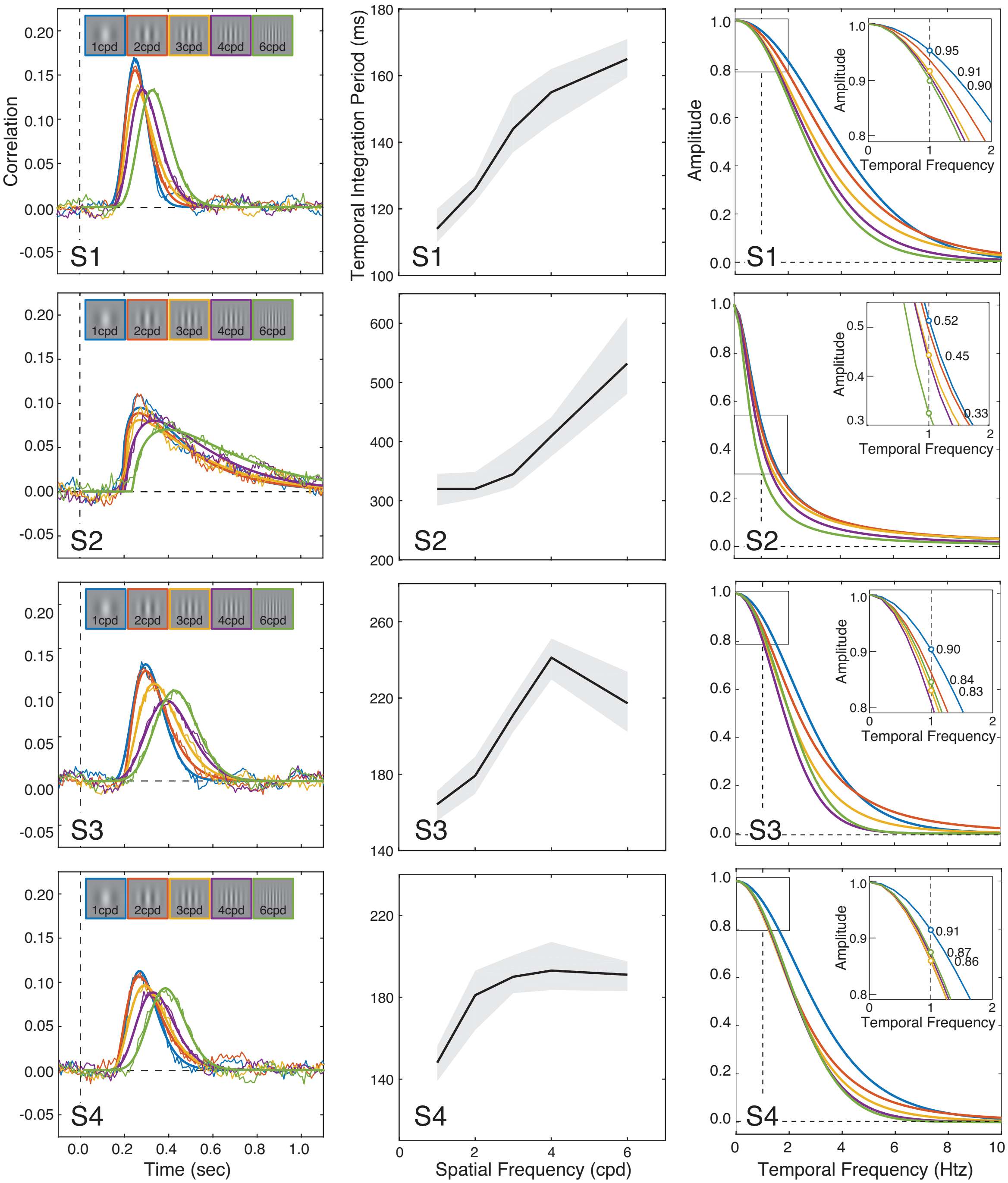

Figure S5. Experiment 3 stimuli and cross-correlograms, full width at half height as a function of spatial frequency, and amplitude spectra for all observers and conditions.

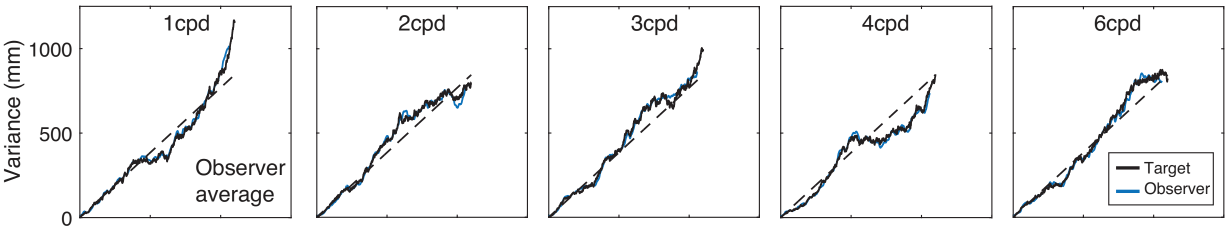

Figure S6. Variance of target positions (black) and variance of cursor positions (blue) (i.e. onscreen distance from the origin) for the mean observer, computed across all runs in each spatial frequency condition. The variances are computed at each time point across all runs. Variance of the target positions across runs increases with time. Targets following random walks tend to drift further from the origin as time passes according to NQ where N is the number of time points and Q is the drift variance on each time step. The variance of the cursor positions tracks that of the target positions. If the sum of the kernel coefficients is greater than 1, then the variance of the cursor positions would be greater than variance of the target positions. If the sum of the kernel coefficients is less than 1, the variance of the cursor positions would be smaller than variance of the target positions. In both cases, the response gain would be different from 1.0, and target tracking would become increasingly inaccurate as the target position drifted farther from the center of the screen. There is no sign of this in the data. Note that in this analysis, the lines indicating cursor position variances have been shifted in time by an amount equal to the subjects' response delays, since cursor positions systematically lag behind target positions. The black dotted line indicates the expected increase in variance. Deviations in the variance of the target positions from the dotted line are due to random sampling and would converge in the limit of an infinite number of runs.
